# Biomarkers of Uremic Cardiotoxicity

**DOI:** 10.3390/toxins13090639

**Published:** 2021-09-10

**Authors:** Bojan Stopic, Sandra Dragicevic, Branislava Medic-Brkic, Aleksandra Nikolic, Marko Stojanovic, Sreten Budisavljevic, Nada Dimkovic

**Affiliations:** 1 Clinical Department for Nephrology, Zvezdara University Medical Center, Dimitrija Tucovica 161, 11000 Belgrade, Serbia; dim@eunet.rs; 2 Institute of Molecular Genetics and Genetic Engineering, University of Belgrade, Vojvode Stepe 444a, 11042 Belgrade, Serbia; sandra.d@imgge.bg.ac.rs (S.D.); aleksni@imgge.bg.ac.rs (A.N.); 3 Department of Pharmacology, Faculty of Medicine, University of Belgrade, Clinical Pharmacology and Toxicology, dr Subotica 1, 11000 Belgrade, Serbia; brankicamedic@gmail.com (B.M.-B.); stojanovic.dr.marko@gmail.com (M.S.); 4 Clinical Department for Cardiovascular Diseases, Zvezdara University Medical Center, Dimitrija Tucovica 161, 11000 Belgrade, Serbia; sretenb@yahoo.com

**Keywords:** cardiovascular event, biomarkers, inflammation, oxidative stress, acute renal injury, microRNAs

## Abstract

Cardiovascular (CV) morbidity and mortality increase along with the progression of chronic kidney disease (CKD). The potential novel biomarkers of cardiotoxicity have been tested with the aim of the early detection of patients at high CV risk, and among them are markers of inflammation, oxidative stress, acute renal injury, and microRNAs. The study analyzed biomarkers in non-dialysis-dependent (NDD; stage 3a–4 CKD) and dialysis-dependent (DD) CKD patients. The prospective cohort study included 87 patients who were followed for 18 months, during which period newly occurred CV events were recorded. Cox regression analysis confirmed serum albumin, urea, interventricular septum thickness diameter (IVST), the use of calcium antagonist, and erythropoiesis-stimulating agent to be significant predictors of CV outcome. No significant difference was observed in biomarkers of inflammation, oxidative stress, acute kidney injury (IL-18, CRP, ferritin, IMA, SOD, NGAL, and KIM-1), and miR-133a, in regards to the presence/absence of CV event, CV death, and left ventricular hypertrophy. Serum albumin, urea, IVST, and the use of calcium antagonist and erythropoiesis-stimulating agents were confirmed to be factors associated with CV events in CKD patients. Apart from traditional risk factors, new research is needed to define novel and reliable biomarkers of cardiotoxicity in CKD patients.

## 1. Introduction

Chronic kidney disease (CKD) has a great impact on global health, as a direct cause of global morbidity and mortality, especially for the occurrence of cardiovascular (CV) diseases, and CKD is recognized as an independent and strong risk factor for their occurrence [1]. CV morbidity and mortality increase along with a decrease in the estimated glomerular filtration rate (eGFR), and the pathophysiological process of CV damage in patients with CKD occurs much earlier, before the onset of end-stage renal disease (ESRD) [2]. Although patients with ESRD have a 20 to 30 times higher risk of CV death compared to the general population, a large number of earlier studies have shown that CV risk is also increased in all CKD stages [3]. The most prevalent CV diseases in CKD are coronary artery disease (CAD), heart failure (HF), peripheral artery disease (PAD), cerebrovascular insult, and left ventricular hypertrophy (LVH). In patients undergoing chronic hemodialysis (HD) treatment, the prevalence of CAD and HF is 36% and 39%, respectively, while the prevalence of LVH is as high as 75% [4,5].

Apart from the traditional risk factors that contribute to increased CV risk, the literature data confirmed that non-traditional risk factors play an important role in the very complex pathophysiology of CV events in the CKD population [6,7]. Therefore, a large number of biomarkers, potential markers of cardiotoxicity, have been tested and used with the aim of early detection of patients at high risk, and among them are markers of inflammation, oxidative stress, and markers of acute renal injury.

Inflammation and oxidative stress are well-known contributors for atherosclerosis, which is among the underlying mechanisms not only for progressive kidney damage, but also for CV comorbidity [8,9,10,11]. As biomarker of inflammation, interleukin 18 (IL-18), has been tested for being a predictor of CV mortality and seems to be a very important indicator of CV-related death in CKD patients [12]. Also, IL-18, as pro-atherogenic cytokine, contributes to arteriosclerosis in CKD patients, and some studies have tried to test it as a potential biomarker of progression of CKD [13,14].

Catalase, glutathione peroxidase, glutathione reductase, and superoxide dismutase (SOD) are among the main enzymatic antioxidants in the human body [15]. Several antioxidant pathways have been altered in CKD, such as reduced erythrocyte SOD activity [16]. Also, some data confirmed that SOD, as a biomarker of oxidative stress, is associated with the occurrence of CV diseases in patients with CKD [17].

Ischemia-modified albumin (IMA) is also one of the novel biomarkers, and its values were altered in conditions of ischemia, inflammation, and high oxidative stress, and, therefore, it has been tested as a potential biomarker for ischemic heart disease [18,19]. According to some studies, IMA can predict mortality in patients with ESRD [20].

Neutrophil gelatinase-associated lipocalin (NGAL) and kidney injury molecule-1 (KIM-1) are most commonly examined from the group of markers of acute kidney injury, and could potentially play a role in the pathogenesis of CKD and the prediction of CV events. It has been described that elevated plasma NGAL in CKD patients may be an independent predictor of future CV events [21]. Also, it has been observed that with an increase in the urinary NGAL, there is an increase in the incidence of ischemic atherosclerotic events in patients with CKD, independently of GFR, albuminuria, and other comorbidities [22]. Recent studies suggest that elevated urinary KIM-1 values may be associated with a higher risk of developing ischemic heart disease, HF, and overall mortality in patients with CKD; therefore, further investigation of this molecule as a potential biomarker of cardiotoxicity deserve attention [23].

Given that previous research has led to conflicting results, the search for new biomarkers is justified. One of the newest are microRNAs (miRNAs). Previous studies showed the association of various miRNAs with CVD pathogenesis, and altered levels of these biomolecules have been observed in patients with LVH, CAD, HF, PAD, and ischemic cerebrovascular insult (ICVI) [24,25,26,27,28,29,30]. The association of particular miRNAs with the occurrence of CVD in patients with CKD is rarely analyzed. The circulating level of miRNA has been altered in patients with CAD and HF, as well as in HD patients with LVH [28,29,30]. However, the expression of miR-133a was not analyzed in different stages of CKD.

Since the aforementioned biomarkers have not been tested together at different stages of CKD, our study aimed to test different biomarkers as potential markers of cardiotoxicity in CKD (stage 3a–4) and ESRD patients. The influence of applied therapy, comorbidity, echocardiographic parameters, and standard laboratory biomarkers were also analyzed.

## 2. Results

Our study included 87 patients who developed CKD mainly due to hypertensive nephroangiosclerosis (50%) and diabetic nephropathy (34.5%). Also, the majority of patients had a diagnosis of arterial hypertension (98%) and diabetes mellitus (41.2%), either as underlying renal disease or comorbidity.

After the 18-month follow-up period, 52 (57.4%) CV events were registered, including 8 (9.2%) CV deaths. The most common event was angina pectoris de novo 14 (26.9%), peripheral arterial disease for 12 (23.1%), angina pectoris non-stabilis for 8 (15.4%), heart failure for 6 (11.5%), myocardial infarction for 3 (5.8%), and cerebrovascular insult for 1 (1.9%).

The clinical characteristics, laboratory results, and applied therapy were compared between patients who developed CV events and those who did not develop CV events (Table 1). The DD CKD patients developed CV events more frequently than the NDD CKD patients (70.8% vs. 31.7%). As compared with patients without CV events, the patients with CV events more frequently used the calcium antagonist and erythropoiesis-stimulating agents, and they had lower hemoglobin and albumin levels, and higher Tnl-Ultra troponin and plasma urea levels.

Table 2 shows the echocardiographic parameters at the beginning of the study, in patients who developed/did not develop CV events. At baseline, the patients who developed a CV event had significantly less-favorable echosonography findings, as follows: significantly higher values of left ventricular mass (LVM), left ventricular mass index (LVMI), left atrium (LA), E/e ratio, left ventricular posterior wall (LVPWT) thickness, interventricular septum thickness (IVST), and significantly lower values of E/A ratio, compared to patients without CV events.

Cox regression analysis showed that serum albumin, urea, IVST diameter, the use of a calcium antagonist and erythropoiesis-stimulating agent proved to be significant predictors of CV outcome (Table 3). For each unit of albumin increase of 1 mmol/L, the probability of CV events decreased by 40% (*p* = 0.001; OR: 0.60, 95% CI 0.446–0.817). For each unit of urea increase of 1 mmol/L, the probability of the occurrence of CV events increased by 1.33 times (*p* = 0.032; OR: 1.33, 95% CI 1.025–1.730). With each unit of increase in IVST diameter of 1 mm, the probability of the occurrence of CV events increased by 3.82 times (*p* = 0.006; OR: 3.82, 95% CI 1.475–9.905).

The patients with calcium antagonists as chronic therapy were 23.33 times more likely to develop a CV event compared to patients who did not use it (*p* = 0.016; OR: 23.33, CI 95% 1792–303,989). The patients with erythropoiesis-stimulating agents as chronic therapy were 99% less likely to develop a CV event (*p* = 0.019; OR: 0.01, CI 95% 0.000–0.493).

To see the influence of newer biomarkers on the occurrence of CV events, we did a comparative sub-analysis that included 51 patients who were at CKD stage 3b–5HD. The general characteristics of the mentioned subgroup are shown in Table 4, and the results of the examined biomarkers are in Table 5. No significant difference was observed in the biomarkers according to presence/absence of a CV event, CV death, and LVH.

## 3. Discussion

This paper analyzed patients with CKD who had or did not have CV events and, at the same time, we put into context various biomarkers, including traditional ones and some of the most recent. Among all the parameters, Cox regression analysis confirmed that only albumin, urea, interventricular septum thickness, the use of calcium antagonists and erythropoiesis-stimulating agents proved to be factors associated with de novo CV events during the 18-month follow-up period.

Most of the literature data during the past decades addressed the problem of cardiovascular morbidity and mortality in patients with stage 5 CKD. However, all the findings that emerged from these studies did not help to improve patients’ outcomes, and the survival of patients remains very high. Then, it became clear that preventive measures should be applied much before stage 5 (ESRD), when the changes are irreversible. Indeed, the authors confirmed that the risk factor for CV morbidity starts very early during CKD progression, being more prominent with stage 3b and lower. Therefore, in recent years, studies have focused on the earlier stages of CKD, at which time preventive measures may yield some promising results.

After the follow-up period, a significantly higher number of CV events was registered in the HD group of patients. In agreement with previous knowledge, significant CV events may be observed from stage 3b, increasing to stage 5HD [2,3]. In this study, with each increase in urea value by 1 mmol/L, the probability of CV events increased by 1.33 times, and this result confirms the clearly defined thesis that uremia is a strong and independent risk factor for CV disease. Therefore, the measures aimed at slowing the progression of CKD are crucial for the reduction in CV complications in CKD patients.

We have shown that patients with CV events had lower hemoglobin and albumin values compared to patients without CV events, and this finding is consistent with early claims that anemia and malnutrition are very important nontraditional risk factors for CV diseases in patients with CKD [31,32]. The role of anemia, as a nontraditional risk factor that contributes to the progression of CKD, development of cardiac hypertrophy, and occurrence and worsening of ischemic heart disease and HF, is well documented [33,34]. At the same time, our finding was that any patient who had erythropoiesis-stimulating agents as therapy for the correction of renal anemia was 99% less likely to develop CV events.

In this paper, we confirmed that, with each increase in albumin value by one gram, the probability for the occurrence of CV events decreased by 40%. Therefore, the prevention and early treatment of malnutrition are of vital importance in the preservation of the CV system in CKD patients [31]. Our finding is in agreement with the previous statements, since a low albumin level is a well-recognized risk factor for CV events and adverse outcomes of dialysis patients. Hypoalbuminemia belongs to Triassic ‘malnutrition, inflammation, and atherosclerosis’ known as MIA syndrome [35]. Numerous studies confirmed that there are two types of hypoalbuminemia, one as a consequence of malnutrition and another associated with inflammation, which is the case in MIA syndrome. The association between malnutrition and adverse cardiovascular outcome in dialysis patients, which stands in contrast to that observed in non-ESRD individuals, has been named “reverse epidemiology” [36]. The explanation for this is precisely inflammation, which is a known risk factor not only for malnutrition, but also for the unfavorable cardiovascular outcome of dialysis patients [35].

Unlike albumin, the role of IMA in the pathogenesis of CV outcome is less analyzed. Albumin is the most abundant protein in human plasma, and acts as a scavenger for divalent metal ions via the N-terminal part of the molecule. In terms of altered albumin, IMA is a form of human albumin in which the N-terminal amino acids are unable to bind to transition metals. IMA formation may occur not only under acute, but also chronic, conditions, such as chronic oxidative stress and inflammation, as observed in dialysis patients. Increased IMA can, in turn, aggravate the severity of oxidative stress and inflammation [37]. In ESRD patients, Sharma et al. found that high IMA levels were correlated with a significantly larger left ventricular size, decreased left ventricular systolic function, and increased mortality [18,19,20]. Recently, it has been shown that high IMA is associated with vascular oxidative stress and inflammation [38,39], and some novel data pointed out that IMA is a good predictive marker of arterial stiffness in hemodialysis patients [40]. Increased arterial stiffness is a frequent finding in hemodialysis patients and is involved in high CV morbidity [41]. Therefore, finding a simple and effective marker of arterial stiffness may help to reduce cardiovascular morbidity in this population. Although no significance was found in the IMA levels between our patients with and without a CV event, with and without LVH, and between survivors and deceased patients, we assume that the role of IMA has to be more closely defined to elucidate its association with particular acute or chronic CV disease and patients’ outcomes.

The present study confirmed that the number of echosonography parameters related to left ventricular enlargement, such as LVM, LVMI, LVPWT, and IVST, was increased in patients with CV events, but only IVST proved to be a potential predictor of CV events in CKD patients. It was already confirmed that LVH is very frequent in CKD patients, even in the pre-dialysis stage. It may predict the worse CV outcome, particularly in ESRD patients [42,43]. We noticed that with each increase in the thickness of the interventricular septum by one mm, the probability of the occurrence of CV events increased by 3.82 times, which is not in agreement with some previous studies, such as study by Cuspidi and associates, where the ability of IVS thickness to predict the occurrence of CV events has not been confirmed, and was an inferior predictor as compared to LVMI [44]. However, this study included the general population, but not patients with CKD, and the data cannot be considered applicable to the CKD patients.

Our study also suggests that the use of calcium antagonists was associated with increased CV morbidity and mortality, and CKD patients who received calcium antagonists were 23 times more likely to develop CV events. Early studies have led to the conclusion that some patients were more likely to develop myocardial infarction if they used fast-acting calcium antagonists to treat arterial hypertension, instead of beta-blockers, and the risk itself increased with increasing the drug dose [45]. The above mentioned has also been confirmed in elderly patients treated for hypertension with short-acting calcium antagonists, who had higher mortality [45]. However, Kestenbaum et al., in a retrospective study, demonstrated that the use of calcium channel blockers decreased the risk of cardiac mortality of ESRD patients by 26%, probably due to the diminishing of LVH, and the normalization of both blood pressure and intracellular calcium concentrations, thus reducing the injury following cardiac arrest [46]. Therefore, this finding needs attention and confirmation in properly designed studies.

The interpretation of elevated myocardial markers of necrosis, such as troponin, in patients with renal failure, is still controversial, since it could be elevated in some patients with CKD even in the absence of signs and symptoms of myocardial ischemia [47]. In our study, there is an increase in Tn1-Ultra troponin values with a decrease in eGFR, and this can be partly explained by decreased residual renal function and by reduced clearance of troponin. Also, so-called uremic cardiomyopathy may contribute to the level of troponin by different mechanisms, including uremia-induced structural changes in the myocardium, endothelial dysfunction, loss of membrane integrity, and ’leaking’ of troponin from cardiomyocytes. Certain studies suggest that troponin is a more prognostic than diagnostic biomarker [48]. In the present study, the patients with registered CV events had higher values of troponin, but its predictive power was not confirmed by Cox regression analysis.

The role of more recent biomarkers (markers of inflammation and oxidative stress, acute kidney injury, and miRNA molecules) in the pathogenesis of CV events were promising, at least on the basis of some reports. The present study found no differences in the values of IL-18, SOD, and IMA between patients with and without CV events, finding that is not in agreement with well-known facts about inflammation and oxidative stress as risk factors for CV disease [49,50]. Also, no difference in AKI markers was observed between the two groups of patients, although previous studies have confirmed the association of KIM-1 and NGAL with certain CV events and death [23]. We believe that a small number of patients and a diversity of serum levels highly influenced the final result. In the present study, we included patients with different eGFR, and perhaps a subanalysis with a larger number of patients by stages would provide more-reliable data.

The literature data on the novel biomarker miR-133a have been followed with enthusiasm. As mentioned, various miRNAs were associated with CVD pathogenesis [24,25,26,27,28,29,30]. Therefore, we included this potential biomarker of uremic cardiotoxicity in our analysis, and we did not find that miR-133a was associated with CV events. Our finding is consistent with the results of Fourdinier et al., who have been studying the levels of miR-126 and miR-223 in patients with CKD. Their study did not show an association between the miRNAs with overall and CV mortality after adjustment for the baseline eGFR [51].

Early studies have shown that miR-133a is important for the regulation of cardiac hypertrophy and the onset of HF [52]. There was no difference in the level of miR-133a between patients with and without LVH. Our findings are not in agreement with the results of previous studies, which indicate a significantly lower level of miR-133a in the group of HD patients with LVH [28,29]. This can be explained by the fact that these studies examined HD patients with LVH, and our study included different groups of CKD patients (stage 3b, 4 and 5HD).

Despite numerous achievements in the field, the mortality of patients with CKD is unsatisfactorily high. Numerous attempts to find newer, more reliable markers of uremic cardiotoxicity that directly affect CV morbidity and mortality have required substantial effort and resources. Still, it seems that ‘classic’ biomarkers (urea, albumin, parameters of LVH) are the most reliable predictor of CV outcome in CKD patients. Therefore, there is still room for new research that would allow us to timely and efficiently prevent CV disease in CKD patients, thus improving their survival.

Our study has several shortcomings, such as a small number of participants and a relatively short follow-up period, and both may be a reason for the less conclusive significance of the evaluated biomarkers. Even so, some well-known markers of CV disease have been confirmed in this paper as well. Unfortunately, the predictive role of the newly investigated biomarker has not been confirmed by this study. Still, this study may offer direction for future research for some new biomarkers, potential cardiotoxins that, either alone or in association with traditional ones, may help in understanding the complex mechanism of CV morbidity and mortality.

In conclusion, serum albumin, urea, IVST, and the use of a calcium antagonist and erythropoiesis-stimulating gens were confirmed to be factors associated with CV events in NDD and DD CKD patients.

## 4. Materials and Methods

### 4.1. Patients

We conducted a prospective cohort study with a follow-up period of 18 months during which newly occurred CV events were recorded. The study included 87 patients treated at the Clinical Department of Nephrology with Hemodialysis, Zvezdara University Medical Center, Belgrade, from March 2017 to September 2018. Inclusion criteria were as follows: age over 18 years, CKD with eGFR less than 60 mL/min/1.73 m^2^ and signed informed consent of the participants. Exclusion criteria were as follows: previous CV events (myocardial infarction, cerebrovascular insult, unstable angina pectoris, peripheral arterial disease, and heart failure), the use of corticosteroids or other immunosuppressive therapy, present active inflammation and malignant disease.

The sample size was calculated based on the reported prevalence of CAD among CKD patients, and the prevalence of CAD in Serbia for the determination of differences between the two proportions for the statistical significance of 0.01 and the power of 80%. Another 15% was added to the sample since we expected the dropout [2,3,4,5,53].

Arterial hypertension is defined by values of systolic blood pressure ≥140 mmHg and/or diastolic blood pressure ≥90 mmHg according to the European Society of Hypertension guideline in 2021 [54].

Hyperlipidemia is defined by elevated values of total cholesterol ≥5.2 mmol/L and/or triglycerides ≥1.7 mmol/L according to the reference values of the local laboratory. Obesity is defined by BMI (body mass index) values greater than 30 kg/m^2^.

According to the level of eGFR, the participants were divided into the following two groups: non-dialysis CKD patients (NDD CKD) stage 3a–4 (eGFR = 59–15 mL/min/1.73 m^2^, no. = 63) and dialysis-dependent CKD patients (DD CKD) (eGFR <15 mL/min/1.73 m^2^, requiring chronic HD treatment, no. = 24). The eGFR was calculated using the MDRD formula (the modification of diet and renal disease) [55]. MDRD study formula was widely adopted to calculate eGFR. The CKD epidemiology collaboration (CKD-EPI) formula improved the accuracy of CKD staging at eGFR ≥ 60 mL/min/1.73 m^2^. Since our study included patients with eGFR less than 60 mL/min/1.73 m^2^, we found the MDRD formula to be appropriate for the staging of CKD in our group of patients [55,56]. All results were presented in subgroups of patients according to the presence/absence of CV event, LVH, and patients’ outcome (survivors and non-survivors).

As CV events are more frequent only starting from stage 3b CKD, we performed the analysis of the biomarker in 51 patients with stage 3b-5HD [2,3].

### 4.2. Registration of a Newly Occurred CV Events

During 18-month follow-up period, each newly occurred CV event was registered including either CV disease or CV death. The following events were recorded: myocardial infarction, new onset or worsening of existing stabile angina pectoris, PAD, HF, and cardiac death. Patients with at least one CV event were classified into the group ’patients with CV events’.

Diagnosis of CV event was based on the clinical examination of patient and data from medical records, as follows:

- Symptoms and signs of heart failure, coronary artery disease, peripheral arterial disease, and cerebrovascular insult,
- Diagnostic procedures: electrocardiography, color Doppler scan, echocardiography, computerized tomography of endocranium,
- Biochemical findings and biomarkers.

### 4.3. Echocardiographic Examination of the Heart

One experienced cardiologist did a standard, transthoracic echocardiographic examination of the heart, and intra-observer variability was 4%. The measurement was conducted on the Toshiba Artida, the SSH-880 CV model of the echo machine following the guidelines of the American Society for Echocardiography [57]. An echosonography examination of the heart in hemodialysis patients was conducted 24 h after the previous hemodialysis (HD) session. LVMI (g/m^2^) was obtained by dividing the obtained value of LVM (g) by body surface area (m^2^). LVMI over 95 g/m^2^ for women and over 115 g/m^2^ for males were criteriums for LVH diagnosis. All patients with echosonographically proven LVH were classified as ’LVH patients’.

### 4.4. Laboratory Analyses

Blood for laboratory analysis for NDD CKD patients was sampled in the morning after 12 h from the previous meal. In dialysis patients, blood was sampled just before dialysis, and after the longest pause from the previous dialysis, and an average of three consecutive measurements is shown. The following parameters were determined using standard laboratory techniques: hemoglobin, calcium, phosphorus, parathyroid hormone, total proteins, albumins, cholesterol, triglycerides, uric acid, ferritin, C-reactive protein, Tnl-Ultra troponin.

### 4.5. Determination of Biomarkers

The tested biomarkers (IL-18, IMA, SOD, and KIM-1) were determined from the serum and urine (NGAL) by ELISA testing (Elabscience ELISA kit: interleukin-18; Elabscience ELISA kit: ischemic-modified albumin; Elabscience ELISA kit: superoxide dismutase; Elabscience ELISA kit: neutrophil gelatinase-associated lipocalin; Elabscience ELISA kit: kidney injury molecule-1). Blood was sampled at the beginning of the study in tubes without anticoagulants, centrifuged at 4 °C for 10 min at 3000 rpm, serum was separated into cryotubes and frozen at −80 °C until testing. C-reactive protein and ferritin were also tested as markers of inflammation using standard laboratory techniques and assays.

For analysis of miR-133a, serum samples were collected into cryotubes and frozen at −80 °C until use. Total RNA was extracted from all serum samples using PureLink viral RNA/DNA mini kit (Invitrogen, Waltham, MA, USA) according to the manufacturer’s protocol. The RNA concentration and purity were determined by UV absorption at 260/280 using spectrophotometer. Reverse transcription was performed using TaqMan microRNA reverse transcription kit (Applied Biosystems, Waltham, MA, USA) according to the manufacturer’s protocol.

Level of miR-133a was measured by quantitative real-time PCR (qRTPCR) using TaqMan miRNA probe (assay ID 002246, Applied Biosystems, Waltham, MA, USA). All analyses were performed in triplicate. For all experiments miR-16 was used for normalization as an internal control (assay ID 000391, Applied Biosystems, Waltham, MA, USA).

Mir-16 is the most frequently used circulating miRNA endogenous control. The inclusion criteria for mir-16 as reference miRNA used in our study were previously reported, and are as follows: (a) detection in all analyzed samples; (b) quantification cycle less than 35, and (c) no significant difference in quantification cycle (Ct) between groups [58,59].

The qRTPCR was performed on 7500 real-time PCR system (Applied Biosystems, Waltham, MA, USA) using the following program: 50 °C for 2 min; 95 °C for 3 min; 40 cycles of 95 °C for 15 s and 60 °C for 1 min.

The value of ΔCt of miR-133a represents the difference in the level of targeted miR-133a and the endogenous control of miR-16. Further, ΔCt was calculated according to the following formula: ΔCt = Ct_miR-133a_ − Ct_miR-16_. There is an inverse correlation between value of ΔCt and serum level of miR-133a (if ΔCt was decreased, the serum level of miR-133a was increased, and vice versa).

### 4.6. Statistical Analysis

Statistical analysis was performed using Statistical Package for Social Sciences 22.0 (SPSS Inc., Chicago, IL, USA). Data were expressed as means ± standard deviation (SD) for continuous variables and percent values for categorical variables. Differences between groups were analyzed using the following: independent sample t-test, analysis of variance test (ANOVA), independent samples Mann–Whitney U test, Kruskal–Wallis test, and Pearson χ2 test. For correlation of variables Spearman correlation test was used. To test the normality of parameters the Shapiro–Wilk test was used. All variables shown as significant were entered in the Cox regression model with CV event as an outcome variable. A *p*-value of less than 0.05 was considered statistically significant.

## Figures and Tables

**Table 1:** General data, baseline clinical characteristics, comorbidities, and therapy in patients with and without cardiovascular events.

	With CV Events, *n* = 37	Without CV Events, *n* = 50	*p* Values
General data and clinical characteristics			
Age (years)	68.2 ± 10.8	66.5 ± 12.5	0.709
Sex (male/female)	24/13	36/14	0.477
Systolic arterial pressure (mmHg)	152 ± 11	147 ± 15	0.249
Diastolic arterial pressure (mmHg)	92 ± 6	92 ± 8	0.313
Body mass index (kg/m^2^)	28.4 ± 3.7	29.2 ± 4.1	0.509
Stages of CKD, n (%):			
- Stage 3a–4 - Stage 5HD	20 (31.7) 17 (70.8)	43 (68.3) 7 (29.2)	0.001
Comorbidities and risk factors, n (%):			
- Stable angina pectoris - Arterial hypertension - Diabetes mellitus - Hyperlipidemia - Smoking - Left ventricular hypertrophy - Obesity (BMI > 30 kg/m^2^)	6 (16.2) 36 (97.3) 13 (35.1) 8 (21.6) 9 (24.3) 20 (52.6) 9 (24.3)	1 (2) 45 (90) 17 (34) 12 (24) 16 (32) 18 (47.4) 17 (34.0)	0.016 0.184 0.912 0.794 0.434 0.063 0.330
Therapy, n (%):			
- ACE inhibitors - Sartans - Calcium antagonists - Beta blockers - Statins - Antiplatelet agents - Erythropoiesis-stimulating agents	26 (70.3) 10 (27) 24 (64.9) 12 (32.4) 4 (10.8) 7 (18.9) 19 (51.4)	36 (72) 8 (16) 14 (28) 12 (24) 7 (14) 8 (16) 13 (26)	0.806 0.209 0.001 0.384 0.658 0.722 0.015
Laboratory parameters			
Hemoglobin (g/dL)	11.2 ± 1.7	12.4 ± 1.8	0.005
Calcium (mmol/L)	2.3 ± 0.1	2.2 ± 0.3	0.492
Phosphorus (mmol/L)	1.2 ± 0.4	1.1 ± 0.3	0.232
PTH (pg/mL)	171 ± 198	92 ± 84	0.105
Total proteins (g/L)	72.8 ± 7.2	72.8 ± 4.5	0.964
Cholesterol (mmol/L)	4.6 ± 1.1	4.7 ± 1.0	0.689
Triglycerides (mmol/L)	2.1 ± 1.5	1.9 ± 1.3	0.622
Uric acid (mmol/L)	369 ± 99	388 ± 95	0.379
Urea (mmol/L)	19.1 ± 8.8	14.3 ± 7.8	0.012
Albumin (g/L)	39.3 ± 3.7	41.5 ± 3.2	0.032
Tnl-Ultra troponin (ng/mL)	0.042 ± 0.057	0.009 ± 0.007	0.003

CV = cardiovascular. CKD = chronic kidney disease. PTH = parathyroid hormone. BMI = body mass index.

**Table 2:** Baseline echosonography parameters in patients with and without cardiovascular events.

ECHO Parameter	With CV Events, *n* = 37	Without CV Events, *n* = 50	*p* Values
Left ventricular mass (g/m)	204 ± 57	181 ± 58	0.034
Left ventricular mass index (g/m^2^)	110 ± 31	94 ± 24	0.025
Left atrium (mm)	41 ± 6	39 ± 6	0.048
Ejection fraction (%)	54 ± 7	53 ± 6	0.678
E/A ratio	0.8 ± 0.3	1.2 ± 1.5	0.013
E/e ratio	11.7 ± 3.2	9.3 ± 2.7	0.002
Left ventricular posterior wall thickness (mm)	10.6 ± 1.4	9.9 ± 1.5	0.039
Interventricular septum thickness (mm)	11.1 ± 1.3	10.1 ± 1.4	0.002

ECHO = echocardiographic, CV = cardiovascular.

**Table 3:** Cox regression analysis of potential risk factors for cardiovascular events.

Variables	OR	CI (95%)	*p*
Erythropoiesis-stimulating agents	0.015	0.000–0.493	0.019
Calcium antagonists	23.338	1.792–303.989	0.016
Left ventricular hypertrophy	0.812	0.079–8.369	0.861
Stable angina pectoris	3.054	0.165–56.504	0.453
Tnl-Ultra troponin	1132.508	0.022–58401936	0.204
Hemoglobin	1.105	0.535–2.281	0.788
Albumin	0.603	0.446–0.817	0.001
Urea	1.331	1.025–1.730	0.032
Ferritin	0.997	0.986–1.009	0.666
Left ventricular mass	0.979	0.943–1.016	0.258
Left ventricular mass index	0.992	0.945–1.042	0.751
Left atrium	1.172	0.954–1.439	0.132
E/A ratio	0.029	0.001–1.349	0.071
E/e ratio	1.162	0.857–1.576	0.334
Left ventricular posterior wall thickness (mm)	0.297	0.078–1.128	0.075
Interventricular septum thickness (mm)	3.822	1.475–9.905	0.006

**Table 4:** General data, baseline clinical characteristics, comorbidities, and therapy in subpopulation of patients in which biomarkers were tested.

	With CV Events, *n* = 25	Without CV Events, *n* = 26	*p* Values
General data and clinical characteristics			
Age (years)	68.1 ± 11.1	65.4 ± 12.6	0.578
Sex (male/female)	14/11	14/12	0.877
Systolic arterial pressure (mmHg)	154 ± 8	153 ± 5	0.498
Diastolic arterial pressure (mmHg)	93 ± 4	94 ± 2	0.060
Body mass index (kg/m^2^)	28.9 ± 3.9	29.1 ± 4.4	0.860
Stages of CKD, n (%):			
- Stage 3a–4 - Stage 5HD	4 (23.5) 13 (76.5)	22 (64.7) 12 (35.3)	0.006
Comorbidities and risk factors, n (%):			
- Arterial hypertension - Diabetes mellitus - Hyperlipidemia - Smoking - Obesity (BMI > 30 kg/m^2^)	25 (50) 13 (42.9) 4 (57.1) 6 (40) 8 (50)	0 (0) 17 (53.3) 3 (42.9) 19 (52.8) 17 (48.6)	0.322 0.461 0.643 0.406 0.925
Laboratory parametars			
Hemoglobin (g/dL)	11.0 ± 1.5	11.8 ± 1.6	0.078
Calcium (mmol/L)	2.3 ± 0.2	2.2 ± 0.1	0.758
Phosphorus (mmol/L)	1.3 ± 0.4	1.2 ± 0.3	0.498
PTH (pg/mL)	154 ± 132	99 ± 84	0.128
Total proteins (g/L)	72.5 ± 5.8	71.9 ± 3.6	0.697
Cholesterol (mmol/L)	4.5 ± 0.9	4.7 ± 0.9	0.370
Triglycerides (mmol/L)	2.2 ± 1.6	1.8 ± 1.2	0.460
Uric acid (mmol/L)	369 ± 111	404 ± 101	0.244
Urea (mmol/L)	20.6 ± 8.2	16.5 ± 7.6	0.075
Albumin (g/L)	39.3 ± 3.7	41.5 ± 3.2	0.032
Tnl-Ultra troponin (ng/mL)	0.042 ± 0.057	0.009 ± 0.007	0.003

CV = cardiovascular. CKD = chronic kidney disease. PTH = parathyroid hormone. BMI = body mass index.

**Table 5:** Biomarkers in patients with and without cardiovascular events.

**Biomarkers**	**With CV Events** **N = 25**	**Without CV Events** **N = 26**	***p* Value**
Ferritin (ng/mL)	217 ± 131	155 ± 126	0.106
C-reactive protein (mg/L)	9.5 ± 19.9	4.4 ± 5.6	0.135
SOD (ng/mL)	3239 ± 16	3235 ± 16	0.277
IMA (ng/mL)	65 ± 95	73 ± 98	0.779
IL-18 (pg/mL)	231 ± 209	190 ± 211	0.383
KIM-1 (ng/mL)	2.3 ± 0.7	2.1 ± 0.8	0.383
NGAL (ng/mL)	1.5 ± 0.5	1.7 ± 0.5	0.301
miR-133a (ΔCt)	6.5 ± 2.0	6.1 ± 2.4	0.611
**Biomarkers**	**Deceased** **N = 7**	**Survivors** **N = 44**	***p* Value**
Ferritin (ng/mL)	241 ± 86	177 ± 135	0.271
C-reactive protein (mg/L)	8.5 ± 7.2	6.6 ± 15.3	0.078
SOD (ng/mL)	3248 ± 12	3235 ± 16	0.092
IMA (ng/mL)	36.5 ± 89.4	75.4 ± 97.2	0.425
IL-18 (pg/mL)	207 ± 271	211 ± 200	0.868
KIM-1 (ng/mL)	2.2 ± 0.7	2.2 ± 0.8	0.868
NGAL (ng/mL)	1.4 ± 0.7	1.6 ± 0.4	0.425
miR-133a (ΔCt)	5.8 ± 2.4	6.4 ± 2.2	0.582
**Biomarkers**	**With LVH** **N = 22**	**Without LVH** **N = 29**	***p* Value**
Feritin (ng/mL)	194 ± 139	168 ± 118	0.559
C-reactive protein (mg/L)	9.6 ± 20.3	4.3 ± 4.4	0.799
SOD (ng/mL)	3238 ± 15	3235 ± 20	0.560
IMA (ng/mL)	79 ± 100	55 ± 89	0.607
IL-18 (pg/mL)	241 ± 217	171 ± 209	0.077
KIM-1 (ng/mL)	2.3 ± 0.7	2.2 ± 0.8	1.000
NGAL (ng/mL)	1.6 ± 0.4	1.6 ± 0.6	0.560
miR-133a (ΔCt)	6.2 ± 2.3	6.3 ± 2.1	0.883

SOD = superoxide dismutase; IMA = ischemia-modified albumin; IL-18 = interleukin 18; KIM-1 = kidney injury molecule-1; NGAL = neutrophil gelatinase-associated lipocalin; CV = cardiovascular; LVH = left ventricular hypertrophy.

## Data Availability

Data sharing not applicable.
